# MiR200-upregulated Vasohibin 2 promotes the malignant transformation of tumors by inducing epithelial-mesenchymal transition in hepatocellular carcinoma

**DOI:** 10.1186/s12964-014-0062-x

**Published:** 2014-10-01

**Authors:** Xiaofeng Xue, Ye Zhang, Qiaoming Zhi, Min Tu, Yue Xu, Jie Sun, Jishu Wei, Zipeng Lu, Yi Miao, Wentao Gao

**Affiliations:** Department of General Surgery, the First Affiliated Hospital with Nanjing Medical University, 300# Guangzhou Road, Nanjing, 210029 China; Department of General Surgery, the First Affiliated Hospital of Soochow University, Suzhou, China

**Keywords:** Vasohibin 2, miR-200a/b/c, Epithelial-mesenchymal transition (EMT)

## Abstract

**Background:**

Hepatocellular carcinoma (HCC) typically relies on tumor transformation and angiogenesis for its malignant behavior, including growth and metastasis. Previously, we reported that Vasohibin2 (VASH2) is preferentially expressed in hepatocellular carcinoma (HCC) tumor tissues and promotes angiogenesis. Here, we further investigated the role of VASH2 in HCC tumor progression.

**Results:**

Bioinformatics analyses and luciferase reporter gene assays confirmed the post-transcriptional regulation of *VASH2* by *miR-200a/b/c*. We then used HepG2 and Hep3B cells, two representative hepatic cancer cell lines, to examine the role of VASH2 in tumors. VASH2 knockdown in HepG2 cells inhibited epithelial-mesenchymal transition (EMT), but VASH2 overexpression in Hep3B cells promoted EMT. Western blot analyses showed that VASH2 promoted EMT through the ZEB1/2 pathway.

**Conclusion:**

VASH2 promoted invasion, reduced apoptosis and increased the proportion of stem cells in vitro and in vivo. These results indicated that VASH2 expression in HCC cells promotes the malignant transformation of tumors by inducing EMT.

**Electronic supplementary material:**

The online version of this article (doi:10.1186/s12964-014-0062-x) contains supplementary material, which is available to authorized users.

## Background

Hepatocellular carcinoma (HCC) accounts for 6% of all cancers worldwide. HCC is the fifth most common malignancy with an estimated half million new cases diagnosed per year globally, and it is the third most common cause of cancer-related deaths [1]. Cytotoxic therapy with PIAF (cisplatin/interferon/doxorubicin [Adriamycin]/5-fluorouracil [5-FU]) is initially promising in many cases of hepatic cancer, but there is a significant increase in toxicity and a lack of a demonstrable survival benefit associated with cytotoxic therapy [2]. Anti-angiogenic agents that inhibit the VEGF pathway have been approved for the treatment of HCC (e.g., Sorafenib for advanced HCC [3]). Unfortunately, less than half of all patients with advanced HCC benefit from these therapies, and these benefits are often only temporary [4]. The poor prognosis of HCC is related to the high likeliness of invasion, metastasis and resistance to radio- or chemotherapy. However, it is still not clear how HCC acquires these malignant behaviors. Thus, it is critical to develop alternative options that target the pathways responsible for the progression of hepatic cancer.

The epithelial-mesenchymal transition (EMT) is a transient and reversible switch from a polarized epithelial cellular phenotype to a fibroblastoid or mesenchymal phenotype characterized by high motility, invasiveness, apoptotic suppression and enhanced extracellular matrix degradation [5-7]. Cells that have undergone EMT are able to transmigrate across basement membranes and stromal tissues as well as to intravasate into the circulatory system, which represents an important step in the biological progression of the malignant transformation of a tumor. Previous reports have demonstrated a novel role of *miR-200* in controlling EMT [8,9]. Increasing evidence has shown that EMT is regulated by the balanced expression of ZEB factors and *miR-200* family members, which are reciprocally linked in the *ZEB/miR-200* feedback loop [10].

Vasohibin 2 (VASH2) belongs to the VASH family, which includes vasohibin 1 (VASH1) and VASH2. Recently, VASH1 was found to be involved in angiogenesis in various solid tumors, and exogenous VASH1 significantly blocks sprouting angiogenesis by tumors [11]. VASH2 was first described by Shibuya et al. [12]. In contrast with VASH1, VASH2 has been found to promote angiogenesis in the process of injury-repairment [13].

Previously, we reported for the first time that VASH2 [14] is a pro-angiogenic factor that is preferentially expressed in hepatic cancer tissues compared with non-cancerous adjacent tissues. Overexpressed VASH2 significantly contributes to tumor growth in vivo and to tumor angiogenesis. In contrast, VASH2 interference attenuates the tumor size and suppresses angiogenesis in a subcutaneous tumorigenesis model. Collectively, our data suggests that VASH2 is responsible for promoting tumor angiogenesis in HCC. The results have been further verified by a follow-up study reporting highly consistent findings [15].

Here, through bioinformatics analyses and preliminary experiments, we further explored the relationship between *VASH2* and *miR-200a/b/c*. Our findings indicated that in HCC, VASH2 is regulated by *miR-200a/b/c* to promote tumor growth, invasion, metastasis and resistance to chemotherapy by inducing EMT in tumor cells.

## Results

### *miR-200a/b* mediates increased VASH2 expression in hepatic cancers

**Figure 1:**
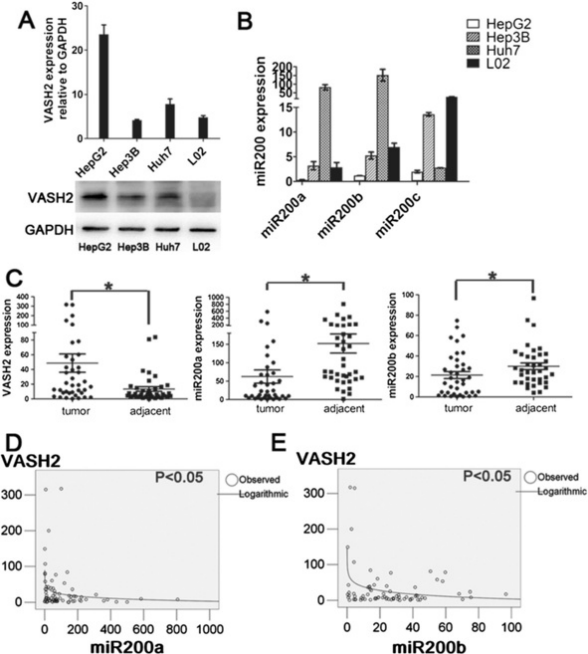
**Expression of VASH2 and miR-200 in cells and HCC samples. (A)** qPCR and western blot of VASH2 in hepatic cancer cells and normal cells. **(B)** qPCR of miR200a/b/c in hepatic cancer cells and normal cells. **(C)** qPCR of VASH2 and miR200a/b in hepatic cancer tissues (*represents p < 0.05). **(D & E)** SPSS analysis of the relationship between VASH2 and miR200a/b.

First, we measured VASH2 transcription and expression in hepatic cancer cells (HepG2, Hep3B and Huh7) and non-cancerous control cells (L02 cells) by qPCR and western blotting. The results (Figure 1A) showed that VASH2 was highly expressed in HepG2 cells but had lower expression levels in the other cell types. We also measured the level of *miR200a/b/c* (Figure 1B) and found that *miR200a/b/c* expression was lowest in HepG2 cells and higher in the other cell types. These results prompted us to measure VASH2 and *miR200a/b/c* expression in hepatic cancer tissues and adjacent tissues. As shown in our previous research [14], *VASH2* expression is higher in hepatic cancer tissues than in the adjacent tissues (p < 0.05). In contrast, *miR200a/b* expression was lower in hepatic cancer tissues than in the adjacent tissues (Figure 1C; p < 0.05), but *miR200c* expression was not different between hepatic cancer tissues and the adjacent tissues (data not shown). The relationship between *VASH2* and *miR200a/b* was analyzed using SPSS software, and a significant logarithmic relationship was found (Figure 1D-E; p < 0.05). Taken together, these results showed that there was a significant relationship between *VASH2* and *miR200a/b* in hepatic cancers where *miR200* may target *VASH2* to increase its expression and function in hepatic cancers.

**Figure 2:**
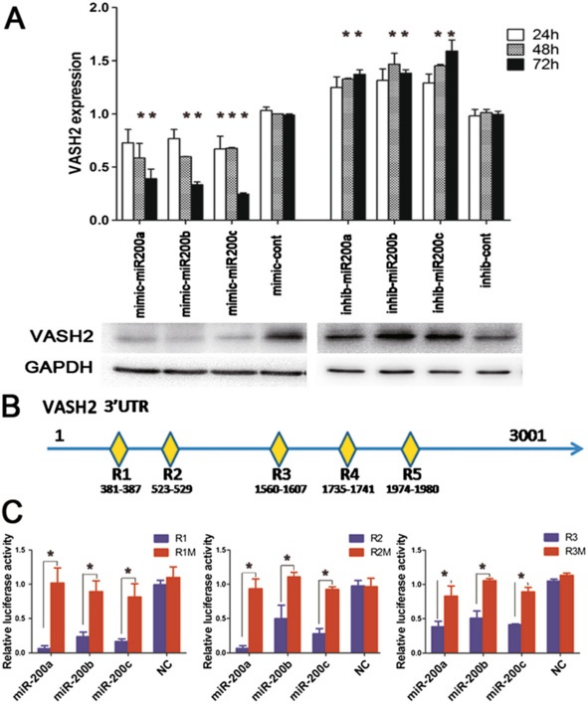
**Transfection of synthetic miR-200 and luciferase reporter gene assay. (A)** qPCR and western blot of VASH2 in HepG2 cells transfected with miR200a/b/c mimics and inhibitors (*represents p < 0.05 compared with the control group). **(B)** Bioinformatics analysis predicted the binding sites of miR200a/b/c in the VASH2 3’UTR (R1-R5 represent 5 separate binding sites). **(C)** VASH2 3’UTR luciferase reporter assay assessing the post-transcriptional regulation of VASH2 by miR200a/b/c (*represents p < 0.05 compared with the mutant group; R1-R5 represents wild-type binding sites; R1M-R5M represents mutant binding sites). miR200a/b/c significantly inhibited luciferase activity in constructs containing the wild-type R1, R2 and R3 binding sites compared with the mutant binding sites, but no effect was observed for constructs containing R4 and R5.

Next, we transfected *miR200a/b/c* mimics and inhibitors into HepG2 and Hep3B cells, and we measured *VASH2* levels by qPCR after 24, 48 and 72 h as well as by western blot after 72 h. The results (Figure 2A) showed that the *miR200a/b/c* mimics down-regulated the level of *VASH2* and that the *miR200a/b/c* inhibitors up-regulated *VASH2* expression relative to the control (p < 0.05), thereby suggesting that *VASH2* is a target of *miR200*. A luciferase reporter gene assay was conducted to confirm this observation. According to the bioinformatics prediction, the *VASH2* 3’UTR has 5 binding sites for *miR200a/b/c* as shown in Figure 2B. The luciferase reporter gene assay showed that *miR200a/b/c* significantly inhibited the luciferase activity in constructs containing the wild-type R1, R2 and R3 binding sites compared with those containing mutant binding sites (Figure 2C), but no effect was observed for constructs containing R4 and R5 binding sites (data for R4 and R5 are not shown). Although the expression of *miR200c* was not different in hepatic cancer tissues compared to adjacent tissues, *miR200c* regulated the level of *VASH2*. These results confirmed that *miR200a/b/c* can directly bind to *VASH2* and mediate *VASH2* expression in hepatic cancers.

### VASH2 induces changes in cell phenotype and EMT markers partly through the ZEB1/2 pathway

**Figure 3:**
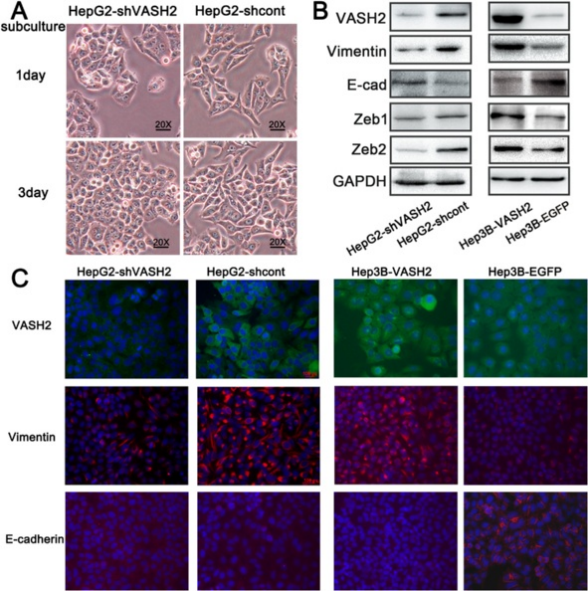
**The effect of VASH2 expression on cell phenotype and marker change. (A)** Cell phenotype changes resulting from different levels of VASH2 expression. **(B)** Western blot analysis showed that the levels of ZEB1/2, an EMT marker and core transcriptional factor, respectively, were affected by changes in VASH2 expression. **(C)** Immunofluorescence (IF) confirmed that the EMT markers changed with VASH2 expression. Magnification of 20 × .

HepG2 cells predominantly exhibit a mesenchymal phenotype. VASH2 knockdown in HepG2 cells caused the cells to change from a spindle-shaped morphology to an epithelioid-like morphology. We examined the HepG2-shVASH2 and HepG2-shcont cells at 1 and 3 days after subculture (Figure 3A). The HepG2-shVASH2 cells acquired the epithelioid-like phenotype and proliferated in clusters, and the HepG2-shcont cells exhibited a spindle-shaped morphology and grew separately. VASH2 overexpression in Hep3B cells did not significantly affect their phenotype (data not shown). We further detected EMT markers, such as vimentin and E-cadherin, in these cells. The results (Figure 3B) showed that VASH2 overexpression increased vimentin expression and decreased the level of E-cadherin. Moreover, VASH2 knockdown decreased vimentin expression and increased the level of E-cadherin. We further detected the core transcriptional factor, ZEB1/2, in EMT by western blot. The results (Figure 3B) showed that ZEB1/2 was upregulated with VASH2 overexpression and downregulated with VASH2 knockdown. Also promoter luciferase reporter gene assay was used to elucidate the mechanism between VASH2 and ZEB1/2. The results (Additional file 1: Figure S1) illustrated VASH2 activated ZEB1/2 promoter activity so that increased ZEB1/2 expression, in consistent with western blot.

In addition, immunofluorescence (IF) was performed to further detect VASH2-induced changes of EMT markers, and the IF results (Figure 3C) were in accordance with the western blot analysis. VASH2 knockdown decreased the level of vimentin fluorescence in HepG2 cells, and VASH2 overexpression upregulated the level of vimentin fluorescence. In contrast, VASH2 overexpression decreased the level of E-cadherin fluorescence. Due to the mesenchymal characteristics in HepG2 cells, we did not detect the epithelial marker, E-cadherin, by IF. Therefore, we confirmed that VASH2 promotes EMT in hepatic cancer cells partly through ZEB1/2.

### VASH2 promotes malignant transformation through EMT

**Figure 4:**
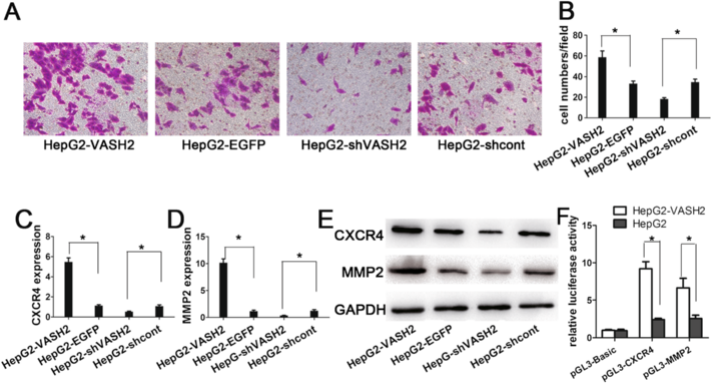
**The effect of VASH2 expression on cell invasion. (A)** Transwell invasion assay in HepG2 cells with different levels of VASH2 expression. **(B)** Cell counts in the Transwell invasion assay. qPCR result for CXCR4 **(C)** and MMP2 **(D)** in HepG2 cells with different levels of VASH2 expression. **(E)** Western blot of CXCR4 and MMP2 in HepG2 cells with different levels of VASH2 expression. **(F)** Promoter luciferase reporter assay for regulation of VASH2 on CXCR4 and MMP2. (*represents p < 0.05 when compared to the control group).

According to previous reports, EMT may promote malignant transformation by increasing the invasion ability and proportion of stem cells in tumors. We conducted Transwell invasion assays to investigate whether VASH2 affected the invasion ability of HepG2 and Hep3B cells. Compared to the control cells, the results (Figure 4A-B) showed that VASH2 overexpression increased the invasive ability of HepG2 cells and that VASH2 interference inhibited the invasive ability of HepG2 cells. VASH2 overexpression also promoted the invasive ability of Hep3B cells (Additional file 2: Figure S2 A-B). Taken together, these data indicated that VASH2 contributes to the invasive ability of hepatic cancer cells. Furthermore, we selectively detected invasion-related genes, such as MMP2 and CXCR4, by qPCR and western blot analyses (Figure 4C-E and Additional file 2: Figure S2C-E). VASH2 overexpression increased the expression levels of MMP2 and CXCR4 in HepG2 and Hep3B cells, but VASH2 knockdown downregulated MMP2 and CXCR4 expression levels. Further promoter luciferase reporter gene assay was used to explain the mechanisms of VASH2 in regulating MMP2 and CXCR4. The results (Figure 4F) suggested VASH2 could definitely upregulate MMP2 and CXCR4 expression. These data suggested that VASH2 might promote the invasive ability by positively regulating pro-invasive genes, such as MMP2 and CXCR4, in hepatic cancer cells.

**Figure 5:**
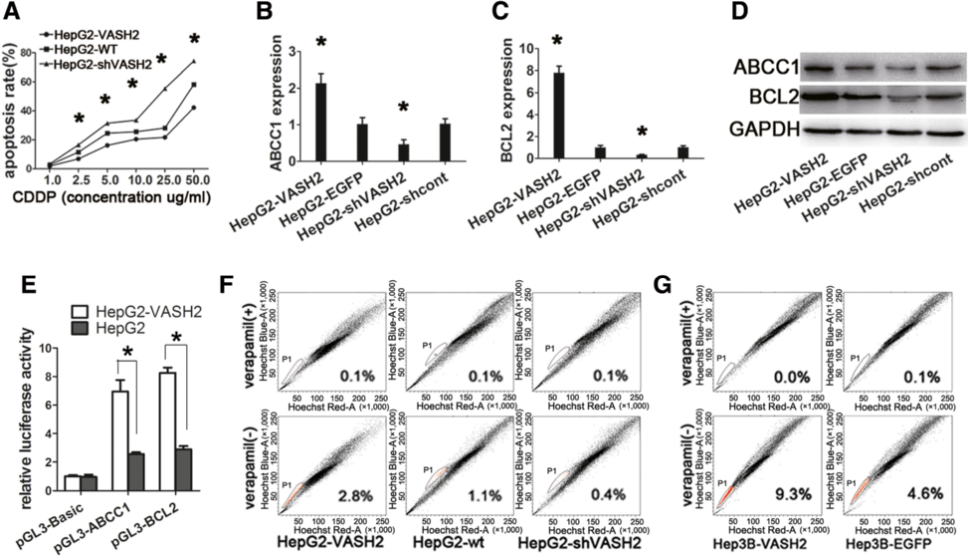
**The effect of VASH2 expression on cell apoptosis and SP cell proportion. (A)** Flow cytometry to determine the apoptosis rate in HepG2 cells with different levels of VASH2 expression after CDDP treatment. Line graph of the apoptosis rate (*represents p < 0.05 when compared to HepG2-wt). qPCR of ABCC1 **(B)** and Bcl-2 **(C)** in HepG2 cells with different levels of VASH2 expression. **(D)** Western blot of ABCC1 and Bcl-2 in HepG2 cells with different levels of VASH2 expression. **(E)** Promoter luciferase reporter assay for regulation of VASH2 on ABCC1 and BCL2. **(F)** SP cell proportion of HepG2 cells with different levels of VASH2 expression. **(G)** SP cell proportion of Hep3B cells with different levels of VASH2 expression. (*represents p < 0.05 when compared to the control group).

We next assessed the effect of VASH2 on the anti-apoptotic ability of hepatic cancer cells. The results (Figure 5A) showed that VASH2 overexpression increased the resistance of HepG2 cells to CDDP treatment, and VASH2 silencing attenuated the anti-apoptotic ability of HepG2 cells upon CDDP treatment. The differences between the groups were significant at a CDDP concentration of 2.5 μg/mL (p < 0.05). Furthermore, the expression of anti-apoptotic genes, such as ABCC1 and BCL-2, was measured by qPCR and western blot analyses (Figure 5B-D). We further did the promoter luciferase reporter gene assay to explain the mechanisms of VASH2 in regulating ABCC1 and BCL-2. The results (Figure 5E) showed VASH2, as a promoting factor, increased ABCC1 and BCL-2 expression. These results suggested that VASH2 increased the anti-apoptotic ability of hepatic cancer cells by upregulating ABCC1 and BCL-2. Besides, we added Hoechst33342 staining to reinforce the anti-apoptosis of VASH2 gene. The result (Additional file 3: Figure S3A) showed that VASH2 overexpression had a more capable resistance to CDDP-induced apoptosis than VASH2 knockdown, which was in consistent with the result of flow cytometry. Furthermore, we tested the relative gene of pro-apoptosis such as Bax, Caspase-3, 6, 9. The result (Additional file 3: Figure S3B) showed VASH2 overexpression gave rise to decrease of pro-apoptotic genes such as Bax, Caspase-3, 6, 9, which elucidated the possible mechanisms of VASH2-induced resistance to apoptosis. Besides, we also measured the relative gene of stem cell property like ALDH1A1, Sox2, Oct4, Nanog. The results (Additional file 3: Figure S3C-D) showed VASH2 upregulated the stem cell marker such as ALDH1A1, Sox2, Oct4, Nanog, which reinforced VASH2 promotion of stem cell proportion.

The SP cell proportion was detected with flow cytometry. The flow cytometry assay (Figure 5F-G) demonstrated that VASH2 overexpression increased the SP cell proportion in HepG2 and Hep3B cells but that VASH2 interference decreased the SP cell proportion in HepG2 and Hep3B cells.

**Figure 6:**
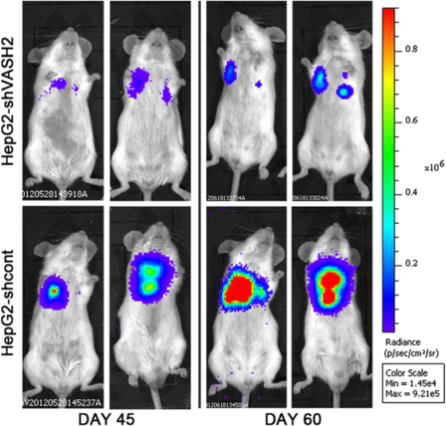
**Bioluminescent imaging of tumors in vivo.** VASH2 interference in HepG2 cells significantly inhibited tumor growth.

We further investigated whether VASH2 promoted invasion in vivo. Tumor cells were injected into the tail vein of SCID mice, and the tumor cells were observed through bioluminescent imaging once a week. The final result (Figure 6) showed that VASH2 silencing significantly inhibited the invasion and metastasis of HepG2 cells. As shown in Figure 6, the HepG2-shVASH2 group presented a lower level of luminescence than the HepG2-shcont group at days 45 and 60. Similarly, VASH2 overexpression promoted the invasion and metastasis of Hep3B cells (Additional file 4: Figure S4). These results confirmed that in addition to positively regulating EMT in vitro, VASH2 promotes tumor invasion and metastasis in vivo.

**Figure 7:**
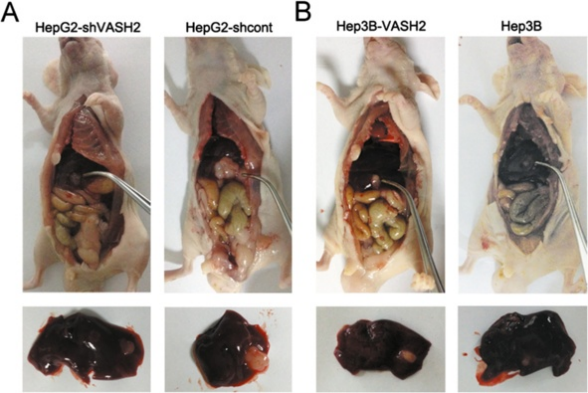
**The orthotopic HCC xenograft model of HepG2 group (A) and Hep3B group (B).**

We also built the orthotopic HCC xenograft model. As the result of the Figure 7, we could see that VASH2 overexpression promoted the tumor growth in vivo. In the contrary, VASH2 knockdown orthotopic reduced the tumor size in vivo. We also measured the orthotopic tumor volume. The result showed HepG2-shVASH2 group vs HepG2-shcont was (18.39 ± 7.87) mm^3^ vs (74.25 ± 24.33) mm^3^, while Hep3B-VASH2 group vs Hep3B group was (20.61 ± 9.98) mm^3^ vs (9.66 ± 2.41) mm^3^. The difference was statistically significant (p < 0.05).

## Discussion

Here, we showed for the first time that VASH2 was preferentially expressed in HCC tumor cells and tissues. Moreover, we showed that *miR-200a/b/c* was expressed at a relatively lower level in tumor cells and that *miR-200a/b* was significantly decreased in HCC tumor tissues. Statistical analyses revealed a negative logarithmic relationship between VASH2 and *miR-200a/b/c*. In vitro experiments demonstrated that *miR-200a/b/c* mimics down-regulated *VASH2* expression and that *miR-200a/b/c* inhibitors up-regulated *VASH2* expression. Luciferase reporter gene assays confirmed that *miR200a/b/c* could directly bind *VASH2* and mediate *VASH2* expression in hepatic cancers. Hence, *miR-200* could negatively regulate VASH2 post-transcriptionally. We showed that VASH2 promoted EMT in HCC cells through ZEB1/2. VASH2 promoted invasion, suppressed apoptosis and increased the proportion of stem cells in HCC. To confirm these results, we investigated the downstream molecular processes by measuring pro-invasion, metastasis and anti-apoptosis gene expression levels.

Despite recent advances in the diagnosis and treatment of HCC, HCC remains a highly lethal disease. The main cause of death in HCC patients is tumor progression with metastasis [16]. There is a considerable body of literature available indicating that hepatocellular EMT is a crucial event in HCC progression, which causes an increase in malignancy of hepatocytes in association with tumor cell invasion and metastasis [7]. Here, we confirmed that VASH2 induced EMT through the upregulation of vimentin and downregulation of E-cadherin in HCC. The results were consistent with the predictions of Sato Y [15]. Of particular importance are ZEB1 and ZEB2, which are crucial regulators of EMT during cancer development [17-19]. These transcription factors activate EMT by binding to E-box elements present in the E-cadherin promoter, thereby suppressing the synthesis of this cell-cell adhesion protein [20,21]. Our results showed that VASH2 may act as an upstream regulator of ZEB1/2 and may affect EMT markers. Furthermore, the EMT phenotype is related to tumor invasion [22], metastasis [23], drug resistance [24-26] and the stem cell proportion [27-29]. In many cancers, such as HCC [30,31], EMT is a particularly important process during early tumor invasion and metastasis. In our studies, by inducing EMT in HCC, VASH2 was shown to promote tumor invasion, metastasis and drug resistance as well as to increase the SP cell proportion. Therefore, we propose that VASH2 may be a candidate target for the treatment of HCC considering its stimulatory effects on EMT and angiogenesis.

In this study, we showed that VASH2, which may represent a novel target for HCC treatment, promoted epithelial cell transformation to the mesenchymal phenotype. During this progression, VASH2 may act as a key transcriptional factor due to its effect on downstream genes. It is necessary to further examine the VASH2 pathway and explore the underlying mechanisms. Hedgehog (Hh) signaling has been reported to induce TGFbeta1 secretion to promote ZEB1 and ZEB2 up-regulation via the TGF-β receptor and NF-γB [32]. Our preliminary results have shown that the Hh pathway may be involved in VASH2. Therefore, it would be interesting to determine the relationship between VASH2 and the Hh pathway with regard to EMT in cancer.

In summary, *VASH2* may be directly mediated by *miR-200a/b* in HCC. Overexpression of VASH2 in HCC induced EMT and thus promoted tumor invasion, metastasis and drug resistance as well as increased the proportion of SP cells. These results indicated that VASH2 may be a molecular target for the treatment of HCC.

## Conclusion

We described in this study the pro-EMT potential of VASH2 in HCC. We showed that VASH2 may be directly up-regulated by miR-200a/b in HCC, thus induced EMT in HCC. Preliminary results showed VASH2 might function through ZEB1/ZEB2 pathway.

## Materials and methods

### Cell culture and HCC samples

The cells were maintained in Dulbecco’s modified Eagle’s medium (DMEM; Gibco, 12100–046, Invitrogen, Carlsbad, CA, USA) containing 10% fetal bovine serum (FBS; Gibco, C2027-050, Uruguay), 100 mg/mL penicillin and 100 mg/mL streptomycin (Gibco, 15140122, Grand Island, NY, USA) at 37°C with 5% CO2. HepG2 and Hep3B human hepatic cancer cells were obtained from the American Type Tissue Culture Collection. Huh7 and L02 cells were provided by Professor Beicheng Sun of the Department of General Surgery, The First Affiliated Hospital of Nanjing Medical University (Nanjing, China). The HCC samples were obtained from Jiangsu Province Hospital (China) in accordance with the Institutional policy. All patients provided written, informed consent which was approved by the ethic committee of Jiangsu Province Hospital.

### Quantitative RT-PCR

Total RNA was extracted from cells and tissues using TRIzol (Invitrogen, 15596–026, Carlsbad, CA, USA), and cDNA was synthesized using the Primescript RT Reagent (TAKARA, DRR037A, Dalian, China). Quantitative RT-PCR was performed on a 7500 Real-Time PCR System (Applied Biosystems) using Taqman probes for GAPDH (Hs99999-m1, Applied Biosystems) and VASH2 (Hs00226928-m1). GAPDH was used as a reference to obtain the relative fold change for targets using the comparative Ct method.

### Western blot

Cell lysates were prepared by extracting proteins with RIPA buffer. The membranes were blocked in 5% non-fat dried milk and incubated overnight at 4°C with the appropriate primary antibodies. The rabbit anti-human VASH2 monoclonal antibody utilized in the western blot analyses was kindly provided by Professor Sato. The antibodies against GAPDH (AG019-1, Beyotime, China), Vimentin (ab8069, Abcam, UK), E-cadherin (ab1416, Abcam), ZEB1 (ab124512, Abcam), ZEB2 (ab25837, Abcam), ABCC1 (ab84320, Abcam), MMP2 (ab86607, Abcam), Bcl-2 (#2870, Cell Signaling Technology, USA) and CXCR4 (ab2074, Abcam) are commercially available.

### Dual luciferase reporter assay

To measure the promoter activity, specific segments of the 5’UTR were amplified and ligated into the pGL3 basic vector. To evaluate the post-transcriptional regulation of the microRNA, sequences from the binding site in the 3’UTR were synthesized and ligated into the pGL3 control vector. Segments surrounding the TSS of the downstream genes (ZEB1/2, CXCR4, MMP2, ABCC1 and BCL2) were amplified and ligated into the pGL3 basic vector. The plasmids were co-transfected with the Renilla luciferase expression plasmid into HepG2 cells. After 48 h, the cells were collected, and the luciferase activity was measured using the Dual Luciferase Reporter Assay System (Promega, E1910, Madison, WI, USA). The relative promoter activity was calculated as firefly fluorescence/Renilla fluorescence.

### Invasion assay

A Matrigel invasion assay was conducted to assess the ability of different groups of cells expressing different levels of VASH2 to penetrate the extracellular matrix (ECM). Cell invasion through the Matrigel was determined using 24-well Corning Transwell chambers (8.0 μm pore size with polycarbonate membrane) (NY, USA, 3412) in accordance with the manufacturer’s instructions. First, the upper chambers were coated with 100 μL of diluted Matrigel (1 mg/mL) (BD company, Bedford, MA, 356243) and incubated at 37°C in 5% CO2 for 3 h. The cells were then trypsinized and suspended in serum-free medium (100 μL) at a concentration of 5 × 104 cells/well and immediately placed into the upper compartment, and 600 μL of DMEM containing 10% FBS was added to the lower compartment. Following a 24 h incubation, the non-invading cells were removed from the upper surface of the membrane by wiping with cotton-tipped swabs. The cells on the lower surface of the membrane were stained with 0.1% crystal violet for 10 min and photographed.

### Bioluminescent imaging

For in vivo imaging, the mice were given the D-luciferin substrate (150 mg/kg in PBS, LUCK-500, Goldbio, MO, USA) by intraperitoneal injection immediately after administration of anesthesia with pentobarbital (50 mg/kg). One minute after administration of the substrate, the anesthetized mice were placed onto the warmed stage inside the camera box. In this study, the animals were imaged for 1 min to ensure consistent photon flux. The mice were imaged weekly for 30 to 60 days until the tumor burden was too great as defined by significant weight loss. The photon measurement was defined around the tumor area and quantified as total photon/s using Living Image software (Xenogen, Corp, Alameda, CA). The tumor size was determined after necropsy at varying time points, and a correlation was made with the average photon emission after live animal imaging.

### HCC orthotopic xenograft model

Following Institutional Animal Ethics Committee permission, four -week-old, male nude mice (BALB/cA-nu (nu/nu)) were purchased from Shanghai Experimental Animal Center (Chinese Academy of Sciences, China). Twenty mice were randomly divided into four groups (HepG2-shVASH2, HepG2-shcont, Hep3B-VASH2 and Hep3B). The orthotopic xenograft tumor model was built as reported by Kyoung Doo Song [33]. A midline incision of the anterior abdominal wall was made, and 2 × 106 cells in a total volume of 0.1 mL of a serum-free medium were directly injected into the left lobe of the liver under anesthesia by pentobarbital sodium. The mice were euthanized after 20 days. The tumor volume was calculated using the formula (width^2^ × length)/2.

### Statistical analysis

All experiments were repeated in triplicate. Where indicated, statistical significance was determined by Student’s t test. P-values less than 0.05 were considered statistically significant.

## Additional files

Additional file 1: Figure S1. Promoter luciferase reporter assay for regulation of VASH2 on ZEB1/2 (*represents p < 0.05). Additional file 2: Figure S2. (A) Transwell assay using Hep3B cells with different levels of VASH2 expression. (B) Cell counts in the Transwell invasion assay. qPCR of CXCR4 (C) and MMP2 (D) in Hep3B cells with different levels of VASH2 expression. (E) Western blot of CXCR4 and MMP2 in Hep3B cells with different levels of VASH2 expression. Additional file 3: Figure S3. (A) Hoechst33342 staining for apoptotic analysis in HepG2 cells with different levels of VASH2 expression after CDDP treatment (White arrow represents apoptotic cells). (B) qPCR measurement of apoptosis-related genes such as Bax, Caspase3, 6, 9 in HepG2 cells with different levels of VASH2 expression. qPCR measurement of stem cell-related genes such as ALDH1A1, Sox2, Oct4, Nanog in HepG2 cells (C) and Hep3B (D) with different levels of VASH2 expression. Additional file 4: Figure S4. Bioluminescent imaging of tumors in vivo. VASH2 overexpression in Hep3B significantly promoted tumor growth.

## Abbreviations

VASH2: HCC Vasohibin2
EMT: Epithelial-mesenchymal transition
