# Supplementary figures and images for: MiR200-upregulated Vasohibin 2 promotes the malignant transformation of tumors by inducing epithelial-mesenchymal transition in hepatocellular carcinoma
Source: Cell Commun Signal. 2014 Oct 1;12:62. doi: 10.1186/s12964-014-0062-x (PMC4195883; doi:10.1186/s12964-014-0062-x)

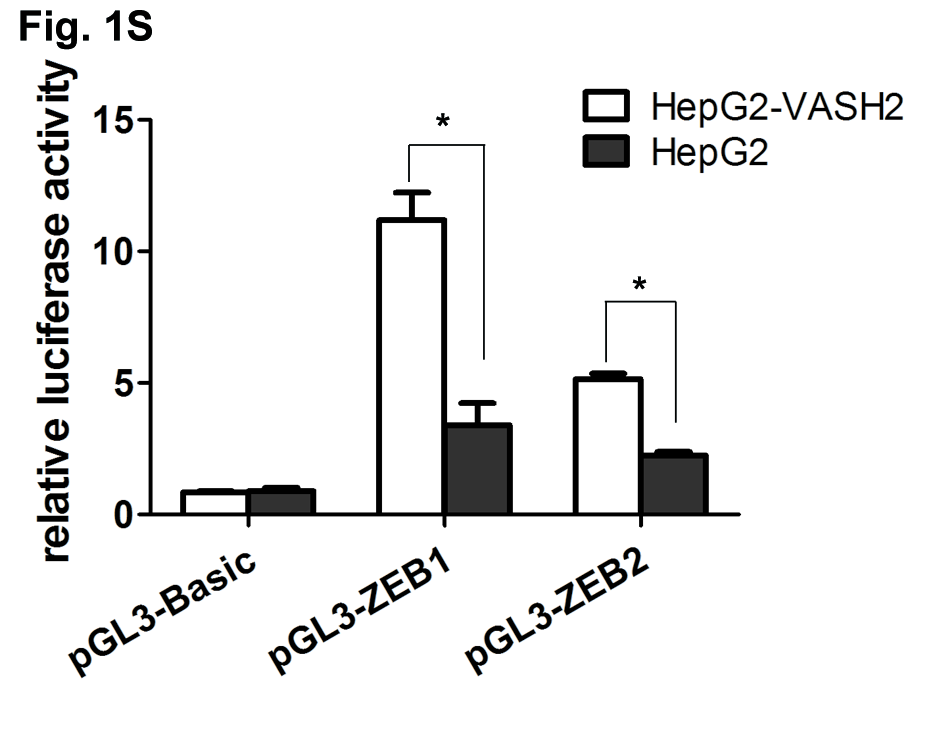

Supplement: Additional file 1: Figure S1. — Promoter luciferase reporter assay for regulation of VASH2 on ZEB1/2 (*represents p < 0.05). [file 12964_2014_62_MOESM1_ESM.tiff]

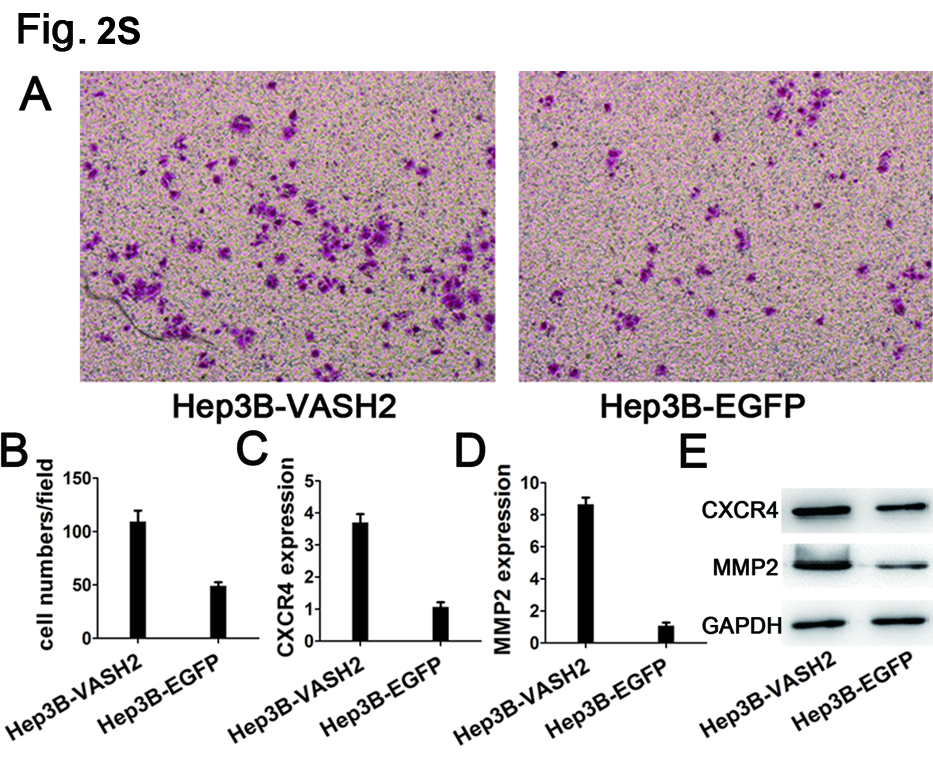

Supplement: Additional file 2: Figure S2. — (A) Transwell assay using Hep3B cells with different levels of VASH2 expression. (B) Cell counts in the Transwell invasion assay. qPCR of CXCR4 (C) and MMP2 (D) in Hep3B cells with different levels of VASH2 expression. (E) Western blot of CXCR4 and MMP2 in Hep3B cells with different levels of VASH2 expression. [file 12964_2014_62_MOESM2_ESM.tiff]

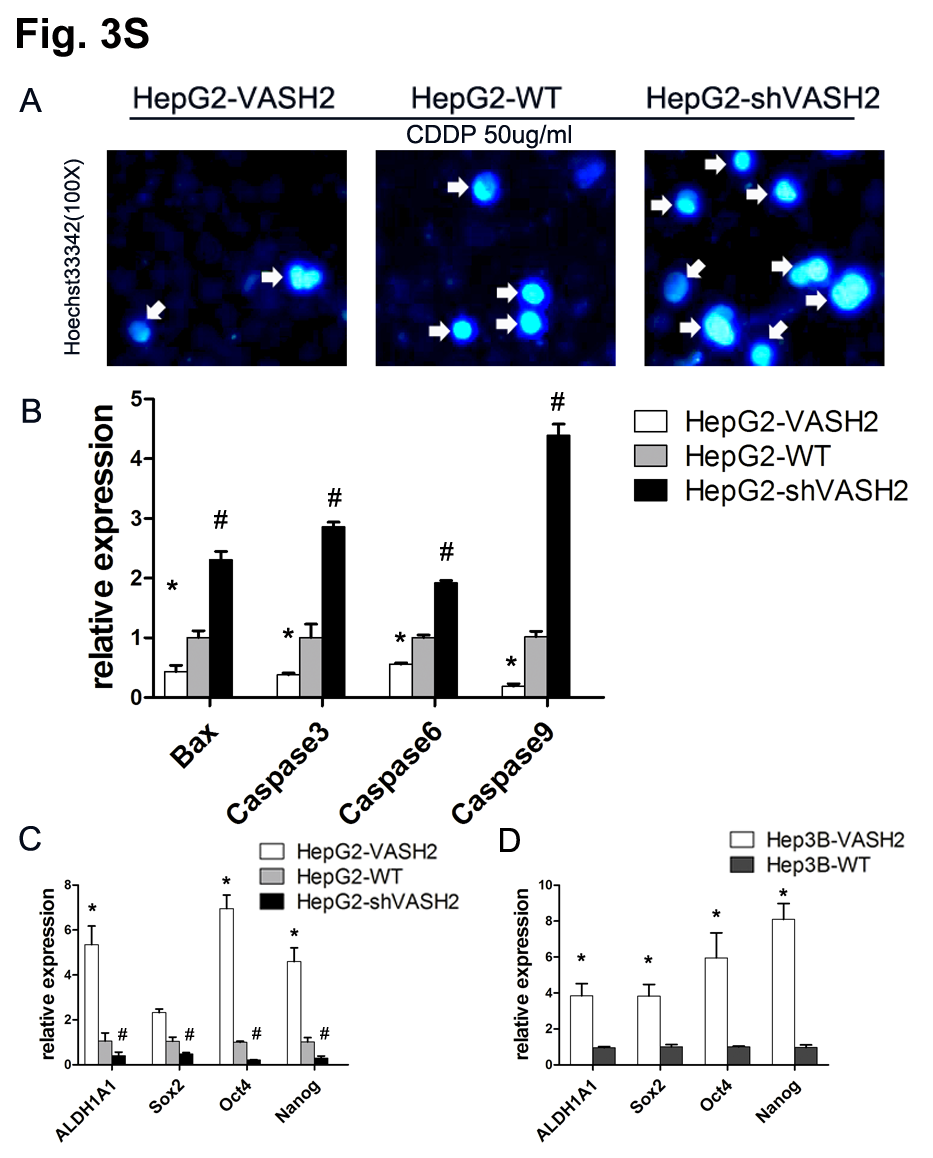

Supplement: Additional file 3: Figure S3. — (A) Hoechst33342 staining for apoptotic analysis in HepG2 cells with different levels of VASH2 expression after CDDP treatment (White arrow represents apoptotic cells). (B) qPCR measurement of apoptosis-related genes such as Bax, Caspase3, 6, 9 in HepG2 cells with different levels of VASH2 expression. qPCR measurement of stem cell-related genes such as ALDH1A1, Sox2, Oct4, Nanog in HepG2 cells (C) and Hep3B (D) with different levels of VASH2 expression. [file 12964_2014_62_MOESM3_ESM.tiff]

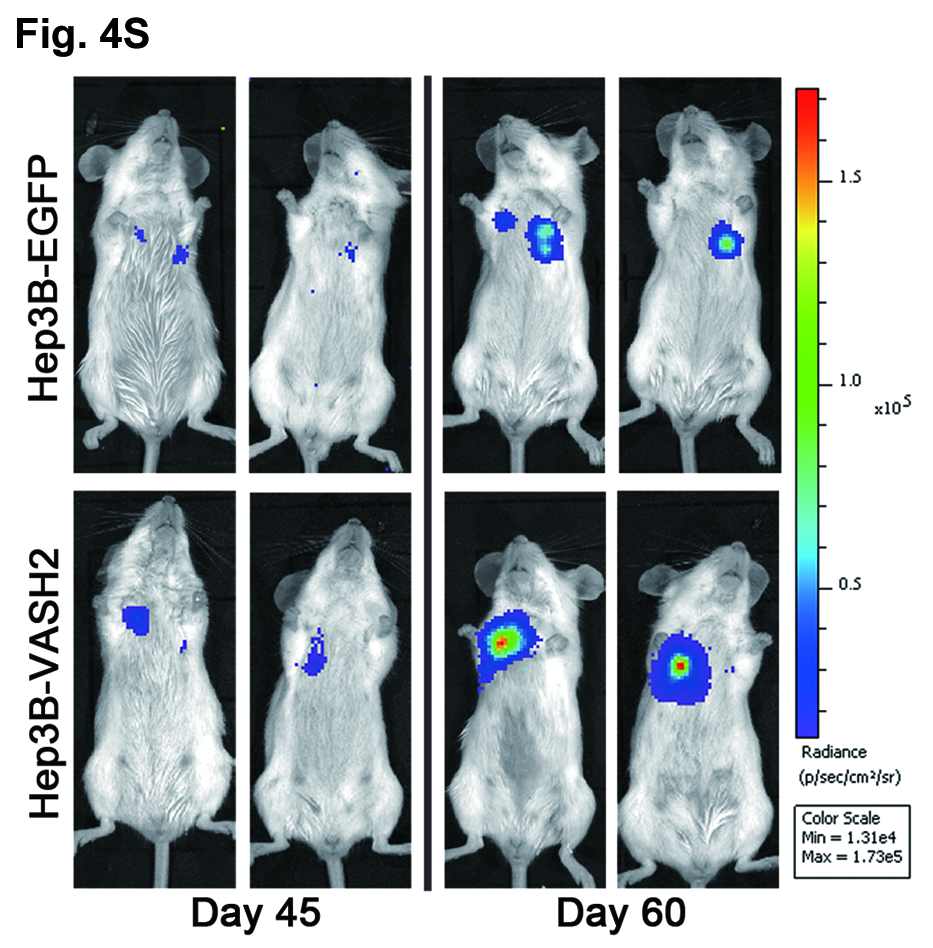

Supplement: Additional file 4: Figure S4. — Bioluminescent imaging of tumors in vivo. VASH2 overexpression in Hep3B significantly promoted tumor growth. [file 12964_2014_62_MOESM4_ESM.tiff]
